# Hypothalamic protein profiling from mice subjected to social defeat stress

**DOI:** 10.1186/s13041-024-01096-4

**Published:** 2024-05-27

**Authors:** Shiladitya Mitra, Ghantasala S. Sameer Kumar, Anumita Samanta, Mathias V. Schmidt, Suman S. Thakur

**Affiliations:** 1 Max Planck Institute of Psychiatry, Kraepelinstr 2-10, Munich, 80804 Germany; 2 CSIR-Centre for Cellular and Molecular Biology, Uppal Road, Habsiguda, Hyderabad, 500007 India; 3 Donders Institute for Brain Cognition and Behavior, Radboud University, Postbs 9010, Nijmegen, 6500GL Netherlands

**Keywords:** HPA axis, Social defeat stress, Hypothalamus, Proteomics

## Abstract

The Hypothalmic-Pituitary-Adrenal axis also known as the HPA axis is central to stress response. It also acts as the relay center between the body and the brain. We analysed hypothalamic proteome from mice subjected to chronic social defeat paradigm using iTRAQ based quantitative proteomics to identify changes associated with stress response. We identified greater than 2000 proteins after processing our samples analysed through Q-Exactive (Thermo) and Orbitrap Velos (Thermo) at 5% FDR. Analysis of data procured from the runs showed that the proteins whose levels were affected belonged primarily to mitochondrial and metabolic processes, translation, complement pathway among others. We also found increased levels of fibrinogen, myelin basic protein (MBP) and neurofilaments (NEFL, NEFM, NEFH) in the hypothalamus from socially defeated mice. Interestingly, research indicates that these proteins are upregulated in blood and CSF of subjects exposed to trauma and stress. Since hypothalamus secreted proteins can be found in blood and CSF, their utility as biomarkers in depression holds an impressive probability and should be validated in clinical samples.

**Supplementary Information:**

The online version contains supplementary material available at 10.1186/s13041-024-01096-4.

## Introduction

Depression is one of the major causes of morbidity worldwide. Depression can arise due to genetic as well as environmental factors. Despite a high rate of prevalence of depression, its causes and proper treatment still remains elusive [1]. In this context, animal models of depression are of interest as they aid in understanding the etio-pathology of depression, finding molecular causes and well as diagnostic methods and screening of drugs. Social defeat model has been designed in a manner that it mimics multiple human situations like bullying, getting beaten up, physical abuse etc. [2]. This make this model an interesting choice to be studied and analysed, results of which may be correlated to human beings.

Multiple brain regions have been studied that may have a profound influence on the pathology of depression. This includes the hippocampus, amygdala, nucleus accumbens, pre-frontal cortex etc. [3]. A study investigating the proteomics changes in the hippocampus associated with susceptibility and resilience in chronic social defeat stress model identified LRP6, NPY and NPY2R as key molecules [4]. In another study, Lipocalin 2 (LCN2) identified again from hippocampus was attributed as a key player in susceptibility in a repeated social defeat stress model [5]. A proteomic study using repeated psycho-social stress model in rats, had identified proteins in the hippocampus belonging to energy metabolism, cytoskeleton, synaptic functions and chaperone activity to be differentially expressed in the stressed group as compared to controls [6]. Interestingly using CSDS model, Guo et al. had shown that ephrin receptor signaling pathway, particularly EPBH6 and Erk pathway to be differentially regulated in the pre-frontal cortex proteome [7]. Thus, while certain molecular changes might be global, different regions of the brain exhibit specific changes in response to stress – particularly social defeat stress.

The Hypothalamic Pituitary Adrenal axis (HPA axis) is at the foremost in response of the animal to stress as it acts as a relay center for the brain and the body. Theoretically, modulation of the HPA axis can make an animal resilient or more susceptible to stress [8]. The expression and release of gluco-corticoids—which are key to stress response, are regulated by the HPA axis [9, 10]. The hypothalamus is the main orchestrator of the HPA axis. While the action of HPA axis when an animal is subjected to stress, is known, interestingly molecular changes in the hypothalamus that might be instrumental in understanding stress response—notably in case when an animal is subjected to social defeat stress, is understudied. The hypothalamus, particularly ventromedial hypothalamus has been shown to be critical to be part of neural circuitry for coping with social defeat stress [11]. Studies have been performed on hypothalamus for investigating differentially expressed genes after social defeat stress [12, 13]. But a proteomics analysis has not been performed and only correlation of genes to proteins had been mentioned in literature. That said, hypothalamus from rats have been subjected to proteomics analysis after Chronic Mild Stress where a subset of genes has been identified and attributed to susceptibility [14]. Similar stress model in mice led to identification of proteins involved in glutamate balance and energy metabolism to be dysregulated in hypothalamic proteome [15]. In a lipo-polysaccharide induced depression model, proteins belonging to Ephrin receptor pathway, Glutamate transmission and AKT pathway was found to be altered in the hypothalamus of stressed mice [16]. But it must be noted that changes in protein levels can differ depending upon the stressor and the intensity of it. It has also been studied in sleep wake cycle [17] and obesity [18] among other conditions. We hope that this study adds further information and aids in understanding of hypothalamic changes under different stressors.

As stated earlier, molecular changes that may happen to the hypothalamus when an animal is subjected to chronic stress may reveal the mode of action of the hypothalamic regions and may also indicate potential bio-markers. Mass spectrometry, particularly iTRAQ based quantitative proteomics has developed into a powerful tool to identify potential bio-marker(s) [19–21]. Hence by employing proteomics to analyse hypothalamus of a mice subjected to social defeat stress, we attempted to find molecular changes that may be extrapolated to depressed human subjects.

## Materials and methods

### Animals used

We used male C57Bl/6 J mice at 3–4 months of age for our experiment. The mice were housed in our in-house facility with 12 h of light and dark cycle. Food was provided ad-libitum. CD1 mice were chosen as the aggressor strain. For this purpose CD1 mice, which were retired breeders, were used. All the experiments were approved by Institutional Animal Ethics Committee (IAEC, CCMB; Reg. No. CPCSEA 20/1999).

### Social defeat experiment

We followed a modified protocol that has been previously described [22, 23]. Briefly, CD1 and C57Bl/6 J mice were housed together with a transparent partition between them. Once a day for 10 min over 10 days, C57Bl/6 J mice were placed in the partition of the CD1 mice (*n* = 10) and were allowed to bully the former. Care was taken that no physical injury was inflicted on the C57Bl/6 J strain. A Latin square approach was followed to prevent habituation.

A separate set of C57Bl/6 J mice (*n* = 7) were not exposed to social defeat served as wild-type controls.

### Behavioral experiment

We performed social avoidance experiment as a test for defeat. The protocol followed has been discussed in other reports [22, 23]. In short, C57Bl/6 J mice were kept in an open field with a transparent grilled enclosure/cage at one side. It was either empty or contained a CD1 aggressor mice. Area around the box was demarcated as interaction zone. Using Noldus Ethovision we tracked the duration and the path of the C57Bl/6 J mice in the interaction zone in the presence or absence of the aggressor. A ratio between the two was calculated as the interaction ratio and was measured as a percentage of total time spent in both conditions.

### Sample collection

Mice were sacrificed the day after the behavioral tests were performed within 3 h of one round of social defeat and hypothalami were collected and flash frozen in liquid nitrogen for further analysis. Social avoidance data showed that interaction ratio of the defeated group to be significantly lower than that of the controls (*p* < 0.01). We selected 7 mice based on interaction ratio from the cohort and randomly assigned 3 mice for proteomics experiment and 4 mice for validation using Western Blotting and real time rtPCR analysis.

### Proteomic experiment

Samples were processed and analysis was carried out using methods described previously [24]. Data are available via ProteomeXchange with identifier PXD005644. Briefly,

iTRAQ 4 plex: Three each of wild-type and social defeat mice were selected and hypothalamus were collected and pooled. We used 0.5% SDS to extract the proteins. After quantification using BCA kit (Thermo Fischer), we used 200ug of protein from both groups to proceed with labelling reaction. The protocol followed was as described by the manufacturer (ABSciex). TCEP (tris (2-carboxyethyl) phosphine) was used to reduce and methyl methanethiosulfonate (MMTS)- a cysteine blocker for reducing the samples. Samples were subsequently digested with trypsin (Promega Cat#:V511A) overnight using 1:20 (w/w) at 37 °C. 100 ug of each sample was used for technical duplicate and labelled with iTRAQ 4plex (ABSciex) reagents as per manufacturer’s protocol. Peptides from wild type samples were labelled with 114 and 115 tags, while those from social defeat samples were labelled with 116 and 117 tags. After labelling, samples were pooled, desalted using C18 spin columns (Pierce®) as per manufacturer’s protocol. The samples were subsequently processed for LC–MS/MS analysis after SCX fractionation using protocol described in a previous study [25].

LC–MS/MS analysis: Samples were analyzed on UPLC (Dionex The UltiMate® 3000 HPLC) interfaced Orbitrap Analyser (Thermo Scientific, Bremen, Germany) or Q-Exactive. Labelled peptides were introduced in a 15 cm long column (EASY-Spray column ES800, 15 cm × 75 µm ID, PepMap C18, 3 µm) and heated to 30° C. Peptides were separated using linear gradient from 2 to 98% of buffer B (95% acetonitrile and 0.1% formic acid) at a flow rate of 300 nl/min. The gradient length was 50 min. Normalized collision energy (NCE) was set to 27 for fragmentation. Precursor ions were selected based on the charge state with priority of 2 + , 3 + and > 3 + . The dynamic exclusion was set as 30 s during data acquisition. The nano source was operated with 2.2 kV and the capillary temperature at 300 °C.

Data analysis: The data was analysed using Proteome Discoverer (Thermo Scientific, Bremen, Germany) software. NCBI fasta (for mouse) database was used to search for peptides. The workflow consisted of spectrum files, spectrum selector, reporter ion quantification and Sequest. Parameters included Methylthio (C), iTRAQ label at N-terminus of peptide. Lysine were used as a fixed modifications, whereas oxidation of methionine (M) and deamination (N/Q) were used as variable modification. The parent and fragment mass error tolerance were set as 20 ppm and 0.2 Da respectively. Using data from runs from both the machines, we acquired a total 77,751 MS/MS spectra and a total of 23,100 sequences were searched. We applied 5% FDR in our analysis and proteins with minimum of 1 unique peptide were considered.

### Real time analysis

cDNA was prepared from the hypothalamus of wild-type and social defeated C57Bl/6 J mice (*n* = 4) using protocol listed elsewhere [26]. Shortly, RNA was extracted using Trizol (Sigma) and 1-Bromo 3-Chloror Propane followed by isopropanol precipitation of aqueous layer. cDNA was prepared using random primers and manufacturer’s instruction (Primescript cDNA synthesis kit, Takara). Real time analysis was performed using Sybr Green (Invitrogen) reagent in ABI HT 4900 machine according the manufacturer’s instructions and with 58℃ as the annealing temperature. The primers used for the experiment are listed below:

**Table:** 

Gene	Forward	Reverse
*Gata1*	TGGGGACCTCAGAACCCTTG	GGCTGCATTTGGGGAAGTG
*Hspe1*	AGTTTCTTCCGCTCTTTGACAG	TGCCACCTTTGGTTACAGTTTC
*Nefm*	ACAGCTCGGCTATGCTCAG	CGGGACAGTTTGTAGTCGCC
*Vim*	CGTCCACACGCACCTACAG	GGGGGATGAGGAATAGAGGCT

### Western blotting

Proteins from hypothalamus from wild type (*n* = 4) and social defeat (*n* = 4) were extracted in RIPA buffer supplemented with Protease Cocktail Inhibitor. 50ug was loaded in an 10% SDS PAGE gel and transferred onto Nitrocellulose membrane using iBlot dry transfer (Invitrogen) according to manufacturer’s recommendations and blotted with antibodies against Neurofilament Medium (NF-M) (Proteintech 20664-1-AP, 1:1000) and Vinculin as housekeeping (Cell Signaling E1E9V, 1:1000). Images were taken with Chemidoc (BioRad).

### Statistical analysis

Behavioral data were subjected to students t-test. Data from real time PCR and Western Blotting were subjected to non-parametric test. Proteomics data was subjected to FDR analysis.

## Results

**Fig. 1:**
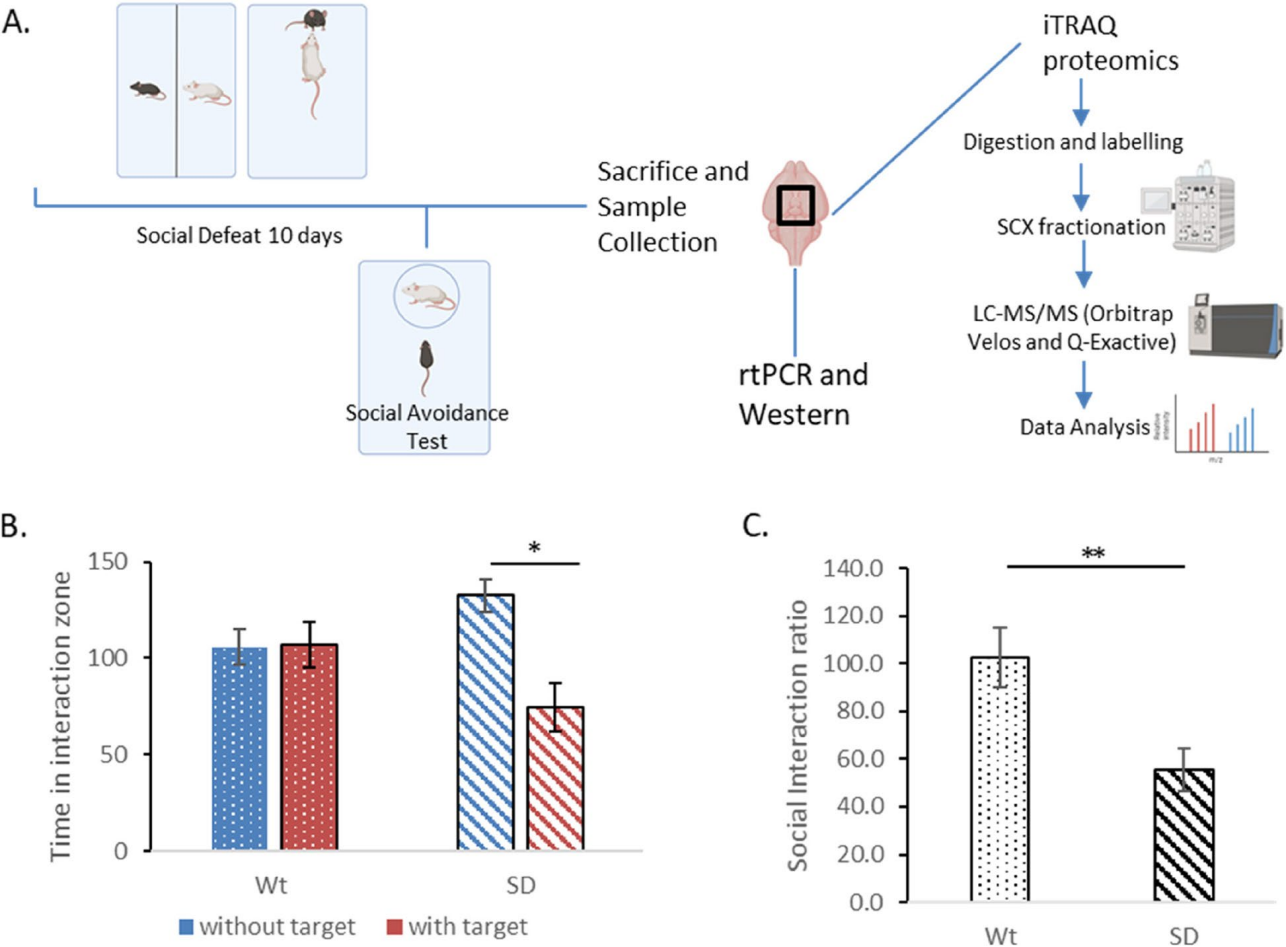
**A** Experimental outline. *Social Defeat Stress procedure*—Male CD1 and C57Bl/6 J are housed in one cage with transparent perforated partition in between. CD1 mice allowed to interact (bully) with the C57Bl/6 J mice for 10 min every day for 10 days. *Analysis of chronic stress by Social Avoidance Test*- C57BL/6 J mice of defeated and control groups are allowed to explore a box containing a target (CD1 mice) in one small perforated grilled cage. The social interaction ratio is calculated as the ratio between time spent in the interaction zone with the target compared to without the presence of the target. *Sample processing and analysis* – Mice were sacrificed and hypothalami were collected and subjected to either iTRAQ based proteomics or validation through rtPCR and Western Blotting. **B** The time spent by the defeated mice (SD) in the interaction zone was significantly lesser (*p* < 0.05) in the presence of a target as compared to without the target. There was significant difference in the time spent in the interaction zone by the unstressed controls (Wt). **C** The social interaction ratio was significantly lesser (*p* < 0.01) in the defeated group (SD) than the unstressed controls (Wt). Graphical figures have been generated by BioRender

The HPA axis is one of the major circuits that play an important role in an organism’s stress response. The hypothalamus is central to the HPA axis’ response to stress [27]. We carried out 4plex iTRAQ quantitative proteomic experiment to study the molecular changes in the hypothalamus when mice were subjected to chronic stress specifically social defeat stress (Fig. 1A).

A total of 17 mice were used for this study. While 7 mice were used as unstressed controls, 10 mice were subjected to social defeat paradigm. Mice subjected to social defeat stress spent significantly less time (*p* < 0.05) in the interaction zone with the presence of the target than without (Fig. 1B). The non-stressed controls did not show any significant differences. Equivalently, the group of mice subjected to social defeat stress showed significantly lesser interaction ratio (*p* < 0.01) as compared to the controls (Fig. 1C).

We used iTRAQ based quantitative proteomics to search for differentially expressed proteins in the hypothalamus of mice after chronic stress. In our analysis, combining runs from both Orbitrap Velos and Q-Exactive, we were able to identify more than 2000 unique proteins (Supplementary Table 1). We found a total of 181 proteins upregulated (> 1.5 folds (116 + 117/113 + 114 or social defeat/unstressed) in the hypothalamus of the mice subjected to social defeat stress as compared to the unstressed controls (Supplementary Table 2). In our analysis we failed to find any major downregulated proteins. The most plausible reason behind this would be technical limitations of our runs. However, it has been reported that after social defeat, the number of genes that were downregulated in the hypothalamus were considerably lesser than those upregulated [13].

**Fig. 2:**
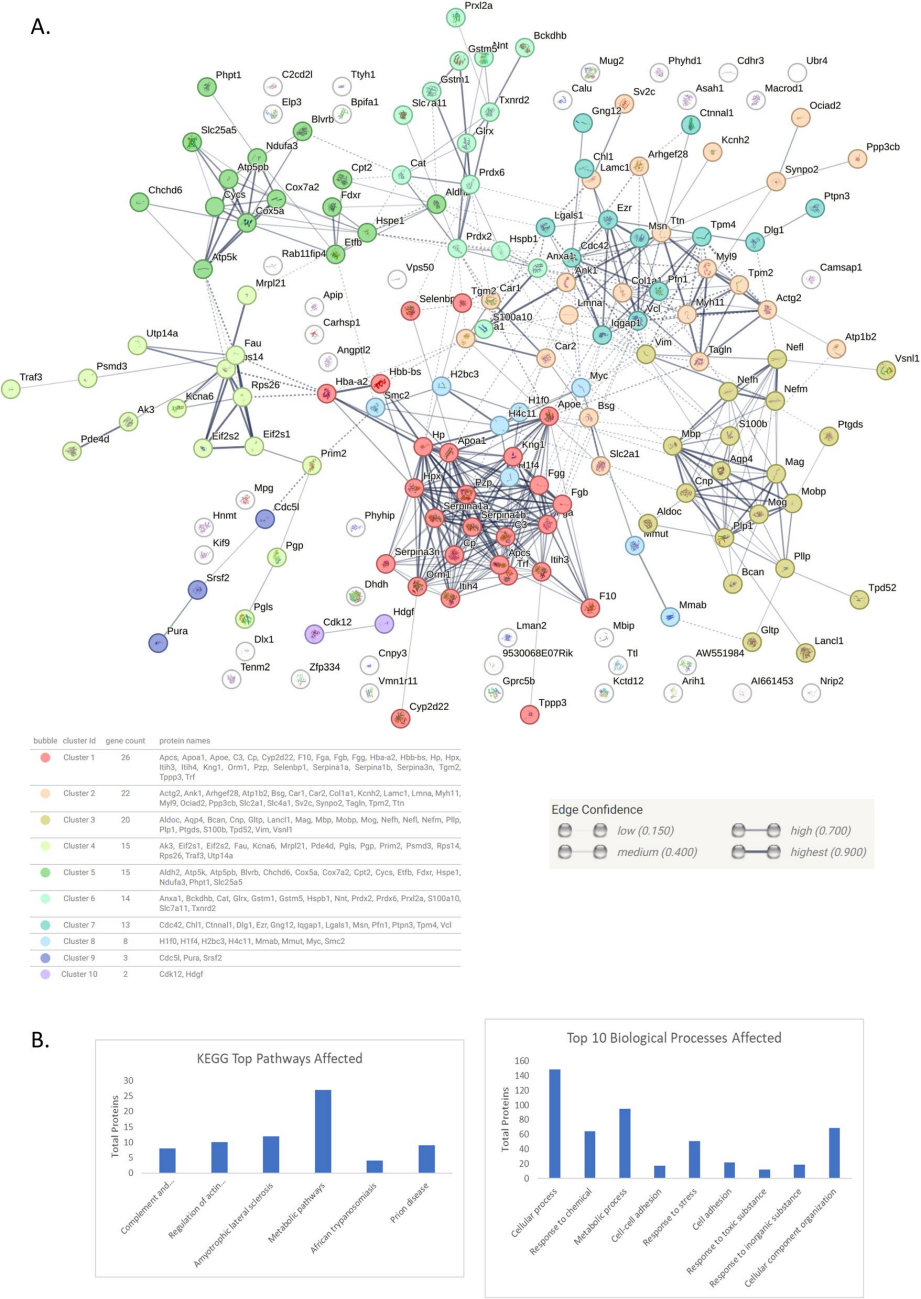
**A** Medium confidence network map of upregulated proteins (SD/Wt)—with thickness of lines depicting increasing confidence (0.15–0.90). k- means clustering with 10 clusters (dotted lines) was applied on these proteins. The network maps have been generated using String v.12. Each color represents a cluster. **B** Top 6 KEGG pathways and top 10 Biological processes affected from analysed proteins. SD: Social Defeat group, Wt: control group

The upregulated proteins were subjected to network analysis using STRING software v 12.0. They were arranged based on KEGG and GO Biological Processes pathway analysis using the same program. The top 6 KEGG pathways and top 10 Biological Processes affected have been depicted in Fig. 2B and in Supplementary Table 3 and 4 respectively. Network analysis was carried out with medium confidence setting with only primary nodes. The proteins were clustered using k-means clustering in 10 prominent interacting groups (Fig. 2, Supplementary Table 5). The groups consisted of the proteins (based on Biological Processes) associated with response to stress and biological regulation (cluster 1), structure and development (cluster 2), nervous system development and neurofilament (cluster 3), translation and metabolic processes (cluster 4), mitochondrial electron transport chain and cellular respiration (cluster 5), redox, ROS and homeostasis pathways (cluster 6), microtubule and cell adhesion (cluster 7), chromatin and nucleosome (cluster 8), spliceosome (cluster 9) and transcriptional regulation (cluster 10).

Since stress might be affecting gene expression at the transcription level, we wanted to check whether upregulation was there at the transcript level of the genes whose protein levels were found upregulated in our study. We selected 4 proteins to check for their respective mRNA levels.

(i) *Gata1* is a transcription factor that has been shown to be associated with depression [28]. Though it was not picked up in our proteomics analysis, we decided to do a qPCR analysis of the same since being a master regulator its change would indicate validation of the model.
(ii) *Hspe1* is subunit of heat shock protein. Heat shock proteins are transcription factors that have been found to be involved in multiple disorders and have been found to be upregulated under stressful conditions [29].
(iii) *Nefm* is one of the three neurofilaments that we indicated in our manuscript can be used as biomarkers for social defeat or trauma induced depression in concurrence with existing literature [30]. We wanted to check if the upregulation of *Nefm* is only at protein level or at mRNA level as well – the latter would indicate that it is transcriptionally upregulated as well.
(iv) Vimentin is an intermediate filament protein that has been shown to play a critical role in stress response [31]. It also interacts with multiple proteins including 14–3-3 which has been shown to be involved in stress adaptation [32]

**Fig. 3:**
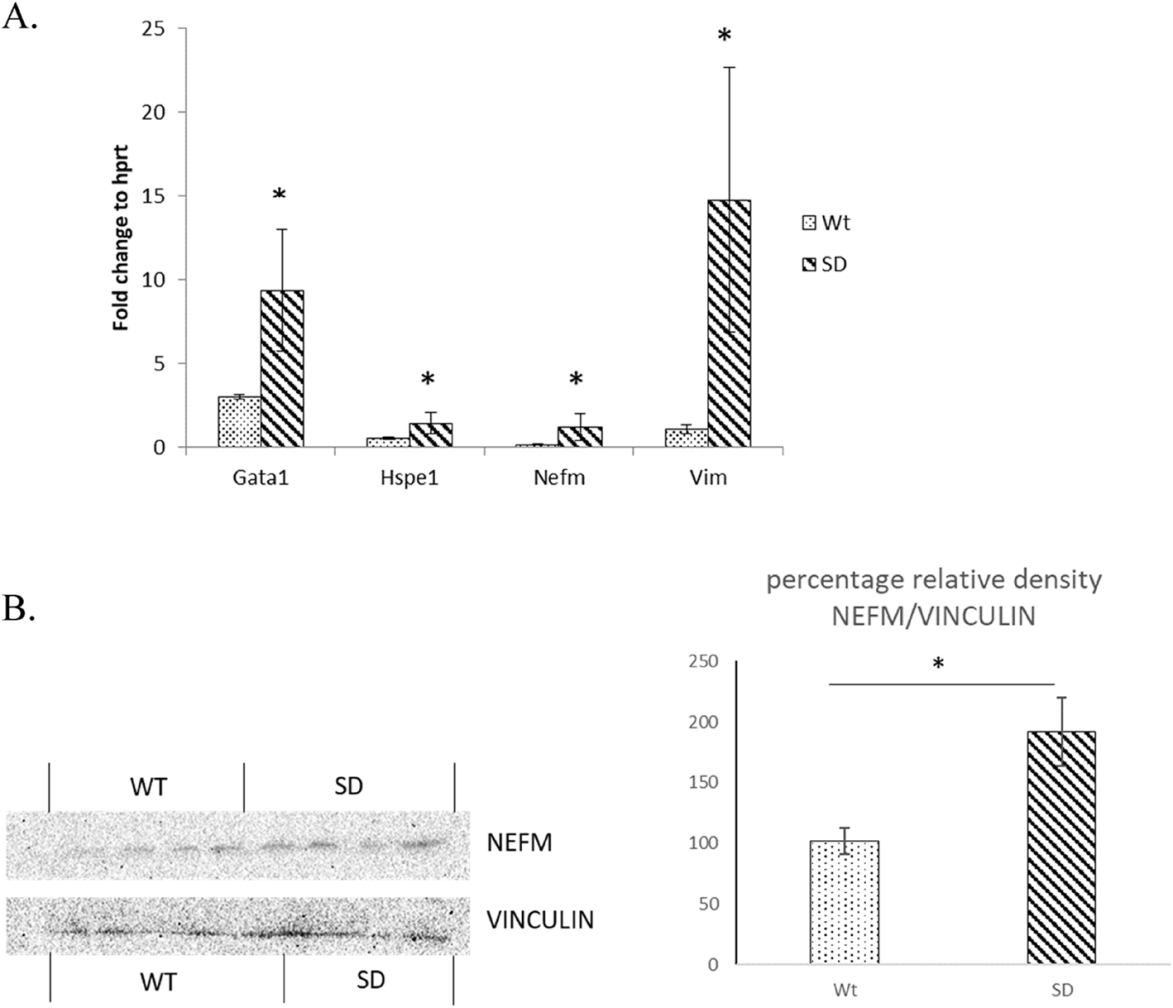
**A** qPCR analysis of genes was carried out for proteins found upregulated. Equivalently transcripts of *Gata1, Hspe1, Nefm* and *Vim* were found to be upregulated (*p* < 0.05) in defeated group (SD) as compared to unstressed controls (Wt); indicating that the effect of social defeat stress might be at the transcriptional level. **B** Western Blotting analysis of levels of NEFM to housekeeping Vinculin and relative density plot showing increased protein levels of NEFM in Social Defeat (SD) group as compared to controls (Wt). * Indicates *p* < 0.05

Interestingly, we found equivalent changes in the transcript levels of *Gata1, Hspe1*, *Nefm* and *Vim* (*p* < 0.05; Fig. 3) thus indicating that the changes at protein levels a result of direct transcriptional regulation. Its noteworthy that protein level changes may not correlate to mRNA level changes, however the reverse has more chances to be true and aids to confidence in the data set generated [33].

To further validate our findings we decided to perform a Western Blotting analysis of one of the key proteins from our data—Neurofilament Medium (NEFM). The result (Fig. 3B) concurred with our proteomics and qPCR data.

## Discussions

Social Defeat induces molecular as well as physiological changes in the brain. Multiple studies have been done to see the effect of chronic stress like social defeat in different brain regions like hippocampus, amygdala, pre-frontal cortex etc. [34–37]. In this present study, we analysed protein profile of hypothalamus, the major constituent of the HPA axis to understand the molecular changes that take place in hypothalamus on subjection to social defeat stress and whether we can correlate them to the response to stress.

A considerable fraction of the proteins found up regulated in the hypothalamus of stressed mice as compared to the unstressed controls belonged to the mitochondrial oxidation–reduction pathway and metabolism. Oxidative stress has been shown to be one of the major players behind pathogenesis of multiple brain disorders like Alzheimers, Parkinson, Huntington and Depression [38, 39]. We found peroredoxin and thioredoxin, both potent anti-oxidants to be upregulated. We also found upregulation in Cytochrome c, which also has anti-oxidant properties. Cytochrome c can bind to super-oxides produced from electron transport chain in mitochondria and neutralise the free radical. Thus, Cytochrome c aids in limiting and regulating the production of oxygen free radicals which may lead to oxidative stress [40]. Similarly, there was an upregulation in the levels of glutathione S-transferases (GST). GST has been known for its role in inactivating electrophilic compounds and for de-toxification [41–44]. Hence upregulation of these anti-oxidants might be a response to the build up of free radicals due to oxidative stress generated due to the chronic stress of social defeat in hypothalamus of mice.

We found upregulation in levels of neurofilaments NEFL, NEFM and NEFH. Transcript analysis indicates that NEFM is also transcriptionally upregulated. Neurofilaments are part of the axo-skeleton and also participate in axonal transport. NF may be accumulated in injury due to mechanical failure of the NF network [45]. In multiple neurodegenerative conditions, NFs acts as a plasma [46] and blood [47] biomarkers for axonal injury and neuronal damage. Interestingly, NEFL has been found to be significantly upregulated in CSF of a concussed boxer even 30 days post-concussion indicating its usefulness as a biological marker for injury and recovery [48]. Taken together, the detection of elevated neurofilament in hypothalamus of mice brain post social defeat stress indicates two points. Firstly, it validates social defeat stress as a model for depression. Secondly, as in the case of concussed boxers, the present study uphold the potential of neurofilaments to be used as biomarkers in several human traumatic/stressful events akin to social defeat model in mice viz., bullying, aggression, subordination and humiliation. Recently few studies have shown that this might be just the case [49–51].

In this study, we observed upregulation of Myelin Basic Protein (MBP) and associated proteins in the brain following stress in accordance with previous studies. MBP has been associated with neuronal death and trauma and has been found to be increased in CSF of dogs post traumatic brain injury [52, 53].

Additionally, upregulation in the levels of several RPS subunits, like RPS14, 26 (a ribosomal protein subunit) were also observed. Ribosomal genes have been found to be upregulated in one more study that analysed de-regulated gene expression in the hypothalamus post social defeat stress [13]. This validates our finding at transcriptional level and also reflects upon the importance of protein translation under stress.

Changes were also found in levels of S100beta, a marker of astrocytes. An increase may indicate towards increased reactive astrocytes as a result of stress [54, 55]. Similar lines hold for microglia, which has already been shown to be increased in social defeat model and in other stress models [56]. An increase in CDC42 found in our proteomic dataset indicated to possible increase in microglial activation as a result of social defeat [57, 58].

We also found increased expression of Fibrinogen alpha, beta and gamma chains along with elevated levels of ApoA1. A complex of alpha–beta Fibrinogen can activate microglia thus leading to neuro-degeneration [59]. Fibrinogen and ApoA1 usually accumulate in the brain from the periphery [59, 60] and their increased levels in hypothalamus might indicate compromised Blood Brain Barrier (BBB) as a result of social defeat stress. In fact recent investigations have shown that leaky BBB might be associated with different regions of brain in mice subjected to stressful paradigms including social defeat and associated with progression of depression like phenotype [61–63]. This might also facilitate the movement of NEFM, NEFL and S100beta into the CSF – the elevated levels of which have been reported in clinical samples from individuals subjected to stress, particularly Traumatic Stress. This re-ignites the debate whether social defeat model is a model for depression like symptoms or ideally a model of PTSD (post-traumatic stress disorder) [64].

Of the different proteins found up regulated, SLC4A1 also deserves special attention as it has been shown to be a promising biomarker in patients with MDD [65].

Several researches have been performed for transcriptomic analysis of different brain regions after social defeat stress. Few of the genes from meta-analysis performed on those studies have been also found in our data set. These include ribosomal genes, heamoglobin genes (Hbb-bs), myelination genes (Mbp), stress and response to insult (Col1a1, Slc25a5) [66, 67]. However, it must be noted that majority of the studies have been performed from hippocampus, prefrontal cortex, amygdala, nucleus accumbens and Ventral Tegmental area, with very few from Hypothalamus and bed nucleus of stria terminalis.

It must be noted that in our study after social defeat stress, we did not segregate the groups into susceptible and resilient populations. The analysis of the proteins can thus be done as a future follow up study after such classification. This can yield further information on protein levels and behavioral outcomes. However, based on our qPCR and western blot analysis, we are confident that at least a subset of the proteins, particularly the neurofilaments show an uniform increase in their levels in socially defeated groups as compare to the controls, thus increasing our confidence in them as potential biomarkers. Interestingly, a subset of the proteins identified in this study including the neurofilament proteins have also been identified to be dysregulated in hypothalamic proteome of rats after chronic mild stress [14].

Taken together, this present study gives information about de-regulated proteins in the hypothalamus of social defeat stressed mice. A large number of changes have been found in multiple studies in different brain regions. We found a considerable proportion of proteins in our data to be similar to those reported in the literature. Changes in hypothalamus are interesting bearing in mind that it interacts directly with the peripheral circulation with modified BBB. We report markers for neuronal death and atrophy and add weightage to the notion that neurofilaments like NEFL, NEFM and MBP may act as potential biomarkers in human stress and traumatic disorders.

### Supplementary Information

Supplementary Material 1.

Supplementary Material 2.

Supplementary Material 3.

Supplementary Material 4.

Supplementary Material 5.

## Data Availability

The mass spectrometry proteomics (raw) data have been deposited to and available at the ProteomeXchange Consortium via the PRIDE partner repository with the dataset identifier PXD005644. The final processed data has been included in the Supplementary Information.

## Abbreviations

HPA: Hypothalamic-Pituitary-Adrenal axis
NF: Neurofilament
RPS: Ribosomal Protein Subunit
BBB: Blood Brain Barrier
MDD: Major Depressive Disorder
CSF: Cerebrospinal Fluid
